# Vitamin D Deficiency and Molecular Changes in Circulating MicroRNAs in Older Adults with Lower Back Pain

**DOI:** 10.1155/2021/6662651

**Published:** 2021-05-17

**Authors:** Hadeel A. Al-Rawaf, Sami A. Gabr, Ahmad H. Alghadir

**Affiliations:** ^1^Rehabilitation Research Chair, College of Applied Medical Sciences, King Saud University, Riyadh, Saudi Arabia; ^2^Departments of Clinical Laboratory Sciences, College of Applied Medical Sciences, King Saud University, Riyadh, Saudi Arabia

## Abstract

**Background:**

MicroRNAs play an essential role in regulating pain processing within a wide range of clinical pain disorders.

**Objectives:**

The present study aimed to evaluate the role of circulating miRNAs as biomarkers of lower back pain in older adults. In addition, the correlation between miRNAs and other related cofounders such as muscle function, adiposity, malnutrition, and Ca and vitamin D intake was assessed.

**Methods:**

A total of 110 older subjects with an age range of 40–60 years were included in this study. The participants were classified according to a modified Oswestry lower back pain disability questionnaire (OSW) into subjects with minimal LBP (*n* = 40; LBP score: 0–20%), moderate LBP (*n* = 35; LBP score: 20–40%), and severe LBP (*n* = 35; LBP score: 41–60%). RT-PCR and immunoassays were used to study the circulating miRNA profile, vitamin D status, and CRP, IL-6, TNF-*α*, s-Ca, s-BAP, s-OC, and s-NTX levels. In addition, malnutrition and muscle performance were estimated in all subjects as other factors related to LBP.

**Results:**

In this study, normal LBP-OSW cutoff values (8.96 ± 3.6) were reported in 36.4% of the total population, whereas 63.6% of the population had higher LBP-OSW scores, classified as follows: 31.8% with moderate LBP (LBP-OSW score: 31.4 ± 9.1) and 31.8% with severe LBP (LBP-OSW score: 54.9 ± 14.6). Four circulating miRNAs, namely, miR-146a, miR-558, miR-155, and miR-124a, as biomarkers of the intensity of back pain were identified in all participants. In subjects with moderate to severe LBP, the expression levels of miR-146a and miR-558 were significantly reduced and those of miR-155 and miR-124a were significantly increased compared to subjects with minimal LBP scores. Subjects with moderate to severe LBP showed a significant increase in adiposity markers, lower PA, muscle performance, malnutrition, and lower Ca and vitamin D intake compared to normal controls. In addition, serum levels of vitamin D and circulated plasma markers of inflammation and bone metabolism such as CRP, IL-6, TNF-*α*, s-Ca, s-BAP, s-OC, and s-NTX were significantly reduced in severe LBP cases compared to those with minimal LBP scores. The expressed circulating miRNAs were significantly associated with the measured muscle performance, adiposity, PA score, inflammation, and bone metabolism cofounders in subjects with higher LBP-OSW scores. The expressed miRNAs, along with other LBP cofounders, were significantly associated with ∼63.9–86.4% of the incidence of LBP in older adults.

**Conclusions:**

In older adults with vitamin D deficiency, the severity of LBP was significantly associated with the expression of circulating miRNAs, adiposity, bone metabolism, inflammation, and muscle performance. In addition, the expressed miRNAs, along with other LBP cofounders, were significantly associated with ∼63.9–86.4% of the incidence of LBP in older adults. These results suggest the possibility of using microRNAs as therapeutics to alleviate established pain and as biomarkers in old adults with painful conditions.

## 1. Introduction

In elderly populations, back pain is the most prevalent source of musculoskeletal soreness worldwide [1]. It was suggested that more than 58% of the elderly suffer from chronic back pain with different variances. This may be due to a lack of concordance in terms of age stratification, definition, and methodology [2–4]. In women, higher rates of lower back pain were reported compared to men [1,3]. The increase in the scores of back pain among women might be due to an increase in bone loss and a rapid decline in bone mineral density (BMD). These parameters collectively result in a greater prevalence of osteoporosis and vertebral fractures in women compared to men [5–7].

In the elderly, the association between lower back pain (LBP) and osteoporosis significantly induces several defects such as skeletal deformities, joint imbalance, and tension in muscular structures with severe or intolerable back pain [8–10]. Patients with lower back pain (LBP) were clinically characterized by tissue damage, muscle weakness, and psychological disturbances, and these consequences were reported in more than 80% of the population who had suffered from lower back pain (LBP) at least once or twice in their life [11,12]. Etiologically, lower back pain is a multifactorial disease with several possible symptoms and causes [13,14]. A variety of factors were shown to be associated with lower back pain, particularly adiposity, chronic comorbidities, poor levels of physical activities, vascular pathology, depression, and psychological disturbances [13,15].

In addition, understanding the role of proper nutrition in the progression and prevention of chronic diseases will allow a better appreciation of the consequences of LBP among younger and older adults [16–18]. Recently, certain dietary interventions have appeared to be of therapeutic benefit in chronic pain management. It was found that low vitamin D and Ca intake was significantly associated with chronic lower back pain, especially in women [19], and that the administration of large quantities of omega-3 polyunsaturated fatty acids greatly mitigated the level of pain, especially in patients with rheumatoid arthritis (RA) [20]. Moreover, it was shown that obesity, malnutrition, and poor eating behavior were highly prevalent in patients with chronic pain [21].

Similarly, cellular molecular changes involved in both inflammatory and bone metabolism processes associated with lower back pain were significantly reported. Indeed, in patients with lower back pain, the levels of tumor necrosis factor-*α* (TNF), interleukin-6 (IL-6), and other related inflammatory markers such as C-reactive protein (CRP) were significantly increased with pain intensity [22–25].

The expression levels of osteocalcin (OC), total calcium (T-Ca), bone-specific alkaline, and N-terminal (NTX) have been reported at different stages of bone resorption and formation processes as bone metabolic biomarkers in both healthy patients and patients with the varying bone loss [26–30].

Although the condition of LBP and its associated consequences are widely documented in the literature [1–30], its etiology, effective management, and prognosis still require further investigations on the basis of changes in the cellular molecular mechanism. Small noncoding inhibitory RNAs known as microRNAs (miRNAs) have been suggested to play a pivotal role in regulating pain processing within a wide range of experimental models and clinical pain disorders [31–34]. In recent studies, the development of diseases was shown to be linked to the aberrant expression of microRNAs [35,36]. Significant changes in the expression of some miRNAs were reported in patients with peripheral inflammation and nerve injury, which both drive severe pain [37–40]. These changes in miRNAs may induce alterations in the expression of some pain-associated genes, leading to an increase in neuronal excitability and behavioral pain hypersensitivity [38,40–42]. These studies indicate that miRNAs might be novel key players in the mechanisms underlying the development and maintenance of chronic pain. In several clinical studies, the possibility of using microRNAs as biomarkers of pain or as therapeutic agents to alleviate established pain was significantly reported in severe painful conditions such as complex regional pain syndrome and fibromyalgia [43–45].

However, the cellular molecular mechanisms of miRNA expression or response in lower back pain and their association with vitamin D status, nutrition, muscle function, and bone metabolism have rarely been studied. Thus, the present study aimed to evaluate the role of circulating miRNAs as noninvasive biomarkers for older adults with lower back pain and to discuss their association with the circulating levels of vitamin D, Ca, inflammation, and bone metabolism markers according to specific degrees of lower back pain. Moreover, the correlation between miRNAs and other related cofounders such as muscle function, adiposity, malnutrition, and Ca and vitamin D intake was assessed.

## 2. Materials and Methods

### 2.1. Subjects

A total of 150 subjects aged 40–60 years were invited to participate in this descriptive cross-sectional study. Considering the exclusion criteria, 110 subjects who had no serious acute or chronic diseases such as chronic renal insufficiency and chronic pancreatitis, cognitive disorders, malabsorption, or a history of osteoporotic fracture were eventually recruited. In addition, subjects with a physical disability or musculoskeletal disorders, who received medications such as steroids that significantly affect body weight, and who received Ca, vitamin D, or multivitamin supplements were excluded from this study.

The study protocol was reviewed according to the ethical guidelines of the 1975 Declaration of Helsinki and approved by the ethical committee of Rehabilitation Research Chair (RRC), King Saud University, Kingdom of Saudi Arabia, under file number ID: RRC-2017-098. Before the collection of data and blood samples, written informed consent was obtained from all participating subjects. Plasma samples were obtained from whole heparinized blood following centrifugation for 1 min at 1400 rpm. All plasma samples were kept frozen at −20 °C until use. The demographic and clinical data of the participants are described in Table 1.

### 2.2. Anthropometric Measurements

Standardized procedures using a tape measure and calibrated Salter electronic scale (Digital Pearson Scale; Adam Equipment Inc., Columbia, MD, USA) were implemented to estimate the height and weight of all participants, respectively [46,47]. Validated universal cutoff values were used to calculate adiposity parameters such as BMI and waist-to-height ratio (WHtR) [46,47]. After the actual body weight and BMI were assessed, the mid-arm circumference (MAC) was measured at the midpoint between the tip of the acromion and the olecranon process on the nondominant side of the body using a flexible tape measure with the patient seated upright with their arm flexed at 90° [48]. A Slim Guide skinfold caliper was used to measure the triceps skinfold, and the mean of three measurements was calculated [49]. The mid-arm muscle circumference (MAMC) was calculated using the MAC and the TSF according to the following standard equation [49]:

(1) $$\begin{array}{c} \text{MAMC}=\text{MAC}\left(\text{cm}\right)-\left(0.314\times \text{TSF}\left(\text{mm}\right)\right). \end{array}$$

### 2.3. Assessment of Lower Back Pain (LBP)

A prevalidated pain-rating numerical scale of 0–10 and a modified Oswestry lower back pain disability questionnaire (OSW) were used to estimate both LBP and pain intensity among subjects [50–52]. LBP was quantified using the OSW scale (a 10-item scale; with scores range from 0 to 100, where higher scores indicate greater disability). According to the OSW scale, subjects with LBP were interpreted as having minimal LBP (LBP score: 0–20%), moderate LBP (20–40%), severe LBP (41–60%), crippling LBP (61–80%), and bedbound or exaggerated LBP (81–100%) [50,51]. Thus, our subjects were classified into three groups: subjects with minimal LBP (*n* = 40; LBP score: 0–20%), moderate LBP (*n* = 35; LBP score: 20–40%), and severe LBP (*n* = 35; LBP score: 41–60%).

### 2.4. Patient Nutritional Assessment

Patients were subjected to a nutritional assessment using a prevalidated subjective global assessment (SGA) score on the day of admission [53,54]. The SGA method has shown good to excellent interobserver reproducibility with good convergent validity [54]. According to the SGA scale, malnutrition scores were classified as follows: well nourished (SGA-A), mild or moderately malnourished (SGA-B), and severely malnourished (SGA-C). Both SGA and body mass index (BMI) were used for malnutrition evaluation [54,55].

### 2.5. Diet Information and Physical Activity

Patients were instructed not to change their normal eating habits during the study period. They were asked to accurately record the amount and type of food, and the fluid consumed using food diaries. Dietary information referred to dietary intake references for physically active people [56,57].

Physical activity was evaluated for seven consecutive days using an ACTi graph GT1M accelerometer (the model was 7164; Fort Walton Beach, FL). The average intensity of PA was calculated from the total number of minutes that each patient participated in sports activities of different intensities [58,59]. This intensity is mainly based on count thresholds and daily activity counts per minute. Subjects with fewer accelerometer counts (≤100 counts/min) were characterized as having a sedentary lifestyle [58,59]. According to their energy expenditure, the PA of all participants was classified as low or sedentary (<4 metabolic equivalents (METs)), moderate (4 METs), and vigorous (7 METs), where 1 MET refers to either an energy expenditure of 1 kcal/kg/h or an oxygen uptake of 3.5 mL/kg/min in a quiet sitting position [60–62]. During the previous month, daily sun exposure was estimated on a weekly basis and divided by 7 to estimate the average number of minutes per day that the patients were exposed to sunlight [60].

### 2.6. Assessment of 25-Hydroxyvitamin D

Serum vitamin 25(OH)D levels were estimated using an ELISA immunoassay [63–65]. A direct competitive chemiluminescence immunoassay with a Liaison auto-analyzer (Liaison, DiaSorin, Turin, Italy) was used to estimate the total 25-hydroxyvitamin (25(OH)D3) concentrations. According to the manufacturer's instructions, serum concentrations of <10 ng/mL were defined as severe VitD deficiency, serum concentrations of <30 ng/mL were defined as VitD insufficiency, and a range between 30 and 100 ng/mL was considered normal [66–68].

### 2.7. Assessment of Bone and Inflammatory Markers

Serum bone markers such as Ca, s-BAP, osteocalcin, and NTX were identified by colorimetric assays using a Cobas Integra® analyzer and different ELISA kits [68–71]. ELISA kits (Hoffmann-La Roche Ltd., Basel, Switzerland) and a MicroVue BAP Immuno-enzymatic assay kit (Quidel Corporation, San Diego, CA, USA) were used to estimate serum Ca and s-BAP concentrations, respectively [68,69]. Serum osteocalcin concentrations were estimated using a MicroVue Osteocalcin enzyme immunoassay kit (QUIDEL Corporation, San Diego, CA) [70]. In addition, the levels of NTX were measured in urine samples using ELISA kits (Osteomark, Ostex International, Seattle, WA, USA), whereas the levels of CRP, IL-6, and TNF-*α* (inflammatory markers) were estimated in the serum samples using Quantikine Human Immunoassay ELISA kits (R&D System, Minneapolis, USA) [71].

### 2.8. Assessment of Circulating miRNAs

#### 2.8.1. Extraction and Purification of Circulating RNA

TRIzol reagent (Clontech Laboratories Inc., Mountain View, CA, USA) was used to extract total RNA from serum according to the manufacturer's protocol. The cDNA of miR-146a, miR-558, miR-155, and miR-124a was then synthesized using the Mir-X miRNA First-Strand Synthesis Kit (Clontech Laboratories Inc.) [72]. The integrity and quantity of total RNA were assessed using an Agilent 2100 Bioanalyzer (Agilent Technologies) [73,74].

#### 2.8.2. Real-Time qPCR of MicroRNAs

Readymade solutions containing the primers and probes for miR-146a, miR-155, and miR-15a (Applied Biosystems, Foster City, CA) were used for real-time RT-PCR carried out with an ABI 7300 system (Applied Biosystems) [72,75]. Quantitative real-time polymerase chain reaction (qRT-PCR) for miR-146a, miR-558, miR-155, and miR-124a was conducted using the Mir-X miRNA qRT-PCR SYBR Kit (Clontech Laboratories Inc.) with the Applied Biosystems 7300 Real-Time PCR System (Applied Biosystems, Foster City, CA, USA) [72–74]. Normalized U6 snRNA levels were used as an internal quantitative control, and the 2^−ΔΔ*Ct*^ system was used to estimate the expression levels of miRNAs used. All reactions were run in duplicate to avoid errors and to accurately determine cycle threshold mean values for each sample including amplified miRNAs [72,73].

### 2.9. Statistical Analysis

The sample size of 110 subjects was selected to give an estimated power of 96% and a significance level of 0.05 with the expected frequency of 9.5%. Statistical software SPSS version (IBM Statistics V.17) was used to analyze the data. Continuous variables were expressed as the mean ± SD, and categorical variables were described as counts and percentages.

The frequency differences between the groups were analyzed using a nonparametric test (Kruskal–Wallis one-way ANOVA) for variables such as lower back pain score, expression levels of miRNAs, VitD deficiency, bone markers, inflammatory markers, muscle performance, and adiposity markers. In addition, multiple stepwise regressions and Pearson's correlation analyses were used to estimate the associations between LBP scores and miRNA levels, vitamin D levels, bone markers, and other related LBP parameters. All tests were two-tailed; because of multiple assessments, results were only considered statistically significant at a *p* value <0.01 [76].

The predictive values of vitamin D status, miRNAs (miR-146a, miR-558, miR-155, and miR-124a), clinical malnutrition assessment score (SGA), physical activity (PA), adiposity (BMI), Ca and vitamin D supplements, and s-Ca, s-BAP, s-OC, CRP, IL-6, and TNF-*α* levels were examined using stepwise linear regression analysis. Variables with the highest *R*^*2*^ value and strong significance were added to the model. Levels of circulating miRNAs (miR-146a, miR-558, miR-155, and miR-124a), bone metabolism markers (s-Ca, s-BAP, and s-OC), inflammatory markers (CRP, IL-6, and TNF-*α*), and vitamin D, as well as Ca and vitamin D intake, SGA, BMI, and MAMC, showed high *R*^2^ values and strong significance. On the other hand, PA, basal metabolic rate (BMR kcal/day), total energy expenditure (TEE, kcal/day), and sun exposure showed low *R*^2^ values and were, thus, neglected in the model.

## 3. Results

**Figure 1:**
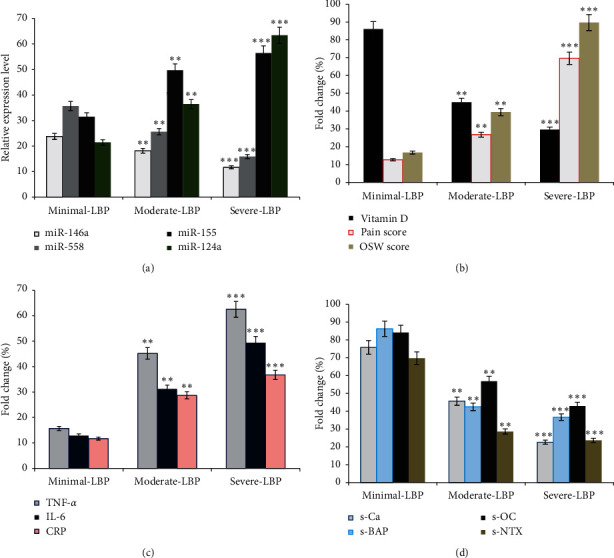
Changes in biological parameters in subjects with different LBP scores. Significant changes were reported in the expression of miRNAs; miR-146a and miR-558 were significantly downregulated and miR-155 and miR-124a were significantly upregulated in subjects with moderate to severe LBP scores compared to subjects with minimal LBP scores (a). A significant decrease in the levels of vitamin D and an increase in pain and LBP-OSW scores were reported in subjects with moderate to severe LBP, compared to those with minimal LBP scores (b). Inflammatory markers as TNF-*α*, IL-6, and CRP were significantly increased in subjects with moderate (*p*=0.01) and severe (*p*=0.001) LBP scores compared to normal controls (c). In addition, biosynthesis of bone metabolism markers as s-Ca, s-BAP, s-OC, and s-NTX was significantly reduced in subjects with moderate (*p*=0.01) and severe (*p*=0.001) LBP compared to subjects with minimal LBP scores (d).

A total of 110 subjects were surveyed for the presence of LBP (Table 1). Approximately, fifty-eight percent of the sample were male (*n* = 64) and forty-one percent of the sample were female (*n* = 46) (Table 1). The incidence of LBP was reported in all subjects (males and females) by using a modified Oswestry lower back pain disability questionnaire (OSW) as reported in Table 1. In total, 36.4% of the subjects had normal cutoff values (LBP-OSW score: 8.96 ± 3.6), whereas 63.6% had high LBP scores, classified as follows: 31.8% with moderate LBP (LBP-OSW score: 31.4 ± 9.1) and 31.8% with severe LBP (LBP-OSW score: 54.9 ± 14.6). This classification was confirmed by the higher pain intensity scores (*p*=0.001) among subjects with moderate and severe LBP compared to those obtained in normal cases (Table 1 and Figure 1(b)).

Anthropometry and muscle mass investigations showed a significant increase in adiposity markers (BMI and WHtR) and lower scores of muscle parameters (MAC, TSF, and MAMC) among subjects with moderate (*p*=0.001) and severe (*p*=0.001) LBP compared to those with minimal LBP (Table 1). In addition, comparable values (*p*=0.01) of the adiposity markers (BMI and WHtR) and lower scores of muscle parameters (MAC, TSF, and MAMC) were reported in female compared to males of the same group of LBPs, whereas females of both moderate (*p*=0.01) and severe (*p*=0.01) LBP showed significant change in BMI, WHtR, MAC, TSF, and MAMC compared to males of the same group (Table 1).

To study the effects of malnutrition and diet (including Ca and vitamin D intake) on the status of LBP, SGA scores were evaluated. Higher SGA scores (B and C) and lower diet scores in terms of inadequate Ca and D intake were significantly (*p*=0.001) reported in subjects with moderate and severe LBP compared to normal subjects (Table 1). In addition, a reduction in PA scores and lower daily exposure to sun were significantly (*p*=0.001) observed among subjects with moderate to severe LBP. Moreover, females showed lower PA scores with a shortage in daily exposure to the sun with higher SGA scores (B and C) and lower diet scores compared to males of the same group (Table 1).

We also estimated the correlation between inflammation and vitamin D status and the incidence of LBP. In this experiment, vitamin D, TNF-*α*, IL-6, and CRP concentrations were estimated in all groups. Significant increases in the levels of TNF-*α*, IL-6, and CRP and a decrease in the level of vitamin D were found in subjects with moderate (*p*=0.001) and severe (*p*=0.001) LBP compared to those with minimal LBP (Table 1 and Figures 1(b) and 1(c)).

The relationship between bone metabolism and LBP was also evaluated in this study. It was found that the biosynthesis of serum bone metabolism markers (s-CA, s-BAP, s-OC, and s-NTX) was significantly reduced in subjects with moderate (*p*=0.001) and severe (*p*=0.001) LBP compared to subjects with minimal LBP, as shown in Table 1 and Figure 1(d). Significant lower changes ((*p*=0.01) in the levels of s-CA, s-BAP, s-OC, and s-NTX were reported in females compared to males of the same LBP group (Table 1).

All subjects with higher LBP showed a lower expression of bone metabolism markers and higher inflammation, which greatly affects physical activity and muscle function.

**Figure 2:**
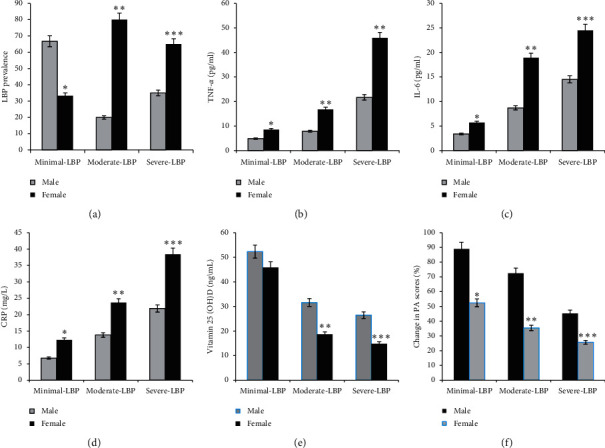
Effect of gender on LBP prevalence rates, serum levels of inflammatory markers as TNF-*α*, IL-6, and CRP, vitamin D serum levels, and physical activity scores in older adults (*n* = 110). Regarding LBP prevalence, a higher incidence of LBP was reported in females compared to males (a). In addition, a significant increase in the levels of TNF-*α*, IL-6, and CRP and a decrease in the levels of vitamin D and PA scores were found in females with minimal (*p*=0.05), moderate (*p*=0.01), and severe (*p*=0.001) LBP compared to males of the same group (b–f).

Regarding gender, females showed high LBP scores compared to males of the same group (Figure 2(a)). In addition, significant increases in the levels of TNF-*α*, IL-6, and CRP and decreases in the level of vitamin D and in PA scores were found in females with minimal (*p*=0.05), moderate (*p*=0.01), and severe (*p*=0.001) LBP compared to male subjects of the same group (Figures 2(b)–2(f)). However, in subjects with minimal LBP, there were comparable levels of TNF-*α* (*p*=0.01), IL-6 (*p*=0.001), CRP (*p*=0.001), vitamin D (*p*=0.001), and PA (*p*=0.001) in females compared to males, as shown in Figure 2 and in Table 1.

**Figure 3:**
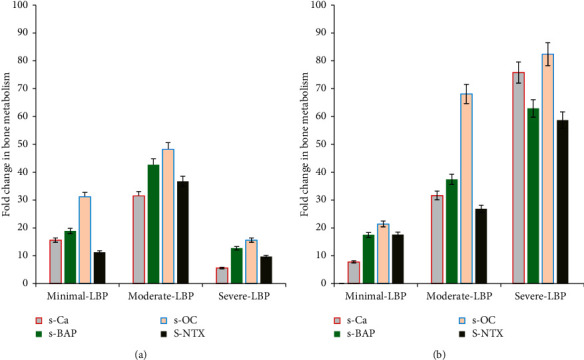
Effect of gender on the biosynthesis of serum bone metabolism markers as s-CA, s-BAP, s-OC, and s-NTX in all subjects with different LBP scores. Fold change in all estimated bone markers showed that s-CA, s-BAP, s-OC, and s-NTX are significantly (*p*=0.001) reduced in female subjects with minimal, moderate, and severe LBP scores compared to male subjects of the same groups.

Similarly, bone metabolism was significantly different in females with LBP scores compared to males. The results showed that the expression levels of s-Ca, s-BAP, s-OC, and s-NTX were significantly (*p*=0.001) reduced in females with minimal, moderate, and severe LBP compared to males of the same groups (Figure 3).

The association of circulating miRNAs with the incidence of LBP and related consequences was estimated using standard RT-PCR techniques in plasma samples of 90 subjects with different LBP scores, as shown in Figure 1(a). The expressions of miR-146a and miR-558 were significantly downregulated in subjects with moderate (*n* = 35, *r* = 0.345, *p*=0.01) and severe (*n* = 35, *r* = 0.315, *p*=0.001) LBP compared to subjects with minimal LBP (Figure 1(a)). On the other hand, the expressions of miR-155 and miR-124a were significantly upregulated in subjects with high LBP scores compared to subjects with minimal LBP (Figure 1(a)). Both up- and downregulated miRNAs correlated positively with BMI, WHtR, muscle function, malnutrition, PA score, vitamin D status, and TNF-*α*, IL-6, and CRP levels, whereas they correlated negatively with the intensity of pain, LBP score (OSW scores), and markers of bone metabolism (s-Ca, s-BAP, s-OC, and s-NTX). In addition, in subjects with LBP, expressed miRNAs showed no significant correlation with gender or age, as shown in Table 2.

The data confirmed that circulating miRNAs (miR-146a, miR-558, miR-155, and miR-124a) can be used as molecular biomarkers separately, in association with other related biological markers such as vitamin D status, adiposity, bone metabolism, and inflammatory markers, or in association with muscle function to predict LBP and its potential risks in older adults. Stepwise regression analysis revealed that miRNAs (miR-146a, miR-558, miR-155, and miR-124a), adiposity markers, SGA, MAC, MAMC, TSF, SGA, TNF-*α*, IL-6, CRP, s-Ca, s-BAP, s-OC, and s-NTX levels, diet scores, and vitamin D deficiency were significantly associated with an incidence of LBP between 63.9% and 86.4% in older adults (Table 3).

## 4. Discussion

LBP was cross-sectionally surveyed in 110 older adults. Only 63.6% of the total population had high LBP scores, classified as follows: 31.8% with moderate LBP (LBP-OSW score: 31.4 ± 9.1) and 31.8% with severe LBP (LBP-OSW score: 54.9 ± 14.6). In general, females showed higher LBP disability scores compared to males.

The expression of circulating miRNAs was shown to be associated with the incidence of LBP and related consequences. The expressions of miR-146a and miR-558 were significantly reduced while those of miR-155 and miR-124a were significantly increased in subjects with moderate and severe LBP scores compared to healthy normal subjects. Both up- and downregulated miRNAs correlated positively with BMI, WHtR, muscle function, malnutrition, PA score, vitamin D status, and TNF-*α*, IL-6, and CRP levels, whereas they correlated negatively with the intensity of pain, LBP score (OSW scores), and markers of bone metabolism (s-Ca, s-BAP, s-OC, and s-NTX).

In the elderly, a link between lower back pain (LBP) and osteoporosis significantly induces several defects such as skeletal deformities, joint imbalance, and tension in muscular structures. These defects are always associated with severe or intolerable back pain [8–10]. Furthermore, lower back pain (LBP) was shown to be associated with severe pain effects at least once or twice in the life of more than 80% of the adult population. Furthermore, LBP showed a significant association with tissue damage, muscle weakness, poor physical activity, and psychological factors [11,12].

Several etiological factors such as adiposity, chronic comorbidities, poor levels of physical activity, vascular pathology, depression, and psychological issues showed a significant association with the incidence of lower back pain [13–15]. Moreover, good nutritional protocols could be used to protect against chronic diseases such as LBP [16–18].

In this study, LBP was shown to be significantly linked with a sedentary lifestyle characterized by poor PA, adiposity, higher scorers of malnutrition, and diets containing inadequate amounts of both Ca and D. Consistent with these data, previous studies have reported a relationship between LBP and PA, obesity, deficient nutrient intake, and poor eating behavior in subjects with LBP [21,77–79]. Moreover, MAC, TSF, and MAMC as parameters of muscle performance were significantly reduced in subjects with moderate and severe LBP compared to those with minimal LBP. The data confirmed that a higher incidence or recurrence of lower back pain among old adults was significantly associated with tissue damage, muscle weakness, and lower muscle performance [11,12,80].

Regarding gender effects on the incidence of LBP, in this study, females with LBP showed a higher VitD deficiency and increased levels of inflammatory markers (TNF-*α*, IL-6, and CRP) compared to males. Moreover, s-Ca, s-BAP, s-OC, and s-NTX as markers of bone metabolism were significantly reduced in LBP females compared to males. The data were significantly correlated with the severity of LBP. The clinical importance of s-Ca, s-BAP, s-OC, and s-NTX bone markers was significantly reported to reflect cartilage and synovium tissue turnover, especially among patients with bone and musculoskeletal diseases such as rheumatoid arthritis (RA), osteoarthritis, and lower back pain [81–83]. A reduction in the expression or biosynthesis of these markers was reportedly exhibited among patients with musculoskeletal diseases, particularly in patients with LBP, and it was found to significantly indicate the degree of joint degradation, which reflects both bone resorption and bone formation among patients [84–86].

Previous studies showed that VitD deficiency could play a role in the reported symptoms of chronic LBP contributing to diffuse pain, bone muscle, and weakness [87]. Low levels of vitamin D produce an increase in the expression of parathyroid hormone, which in turn leads to an increase in bone turnover, thus increasing the risk of microfractures in the endplate or more severe bone loss in females compared to males [88]. A link between lower back pain (LBP) and osteoporosis (OP) or bone loss has been significantly identified in large population studies, especially in the elderly [8,9,89,90]. On the other hand, in elderly persons with bone loss, a reduction in the number and size of muscle fibers with a preferential loss of type II fibers was reported [10], whereby these biosynthetic changes significantly increase the risk of muscle atrophy, bone loss, and bone fractures [91,92].

In this study, like many others, metabolic and inflammatory responses were shown to be associated with muscle damage and injury present in subjects with LBP. Selected inflammatory biochemical mediators (i.e., cytokines as TNF-*α*, IL-6, and CRP) were found to be higher in female subjects with LBP compared to males [93–97].

Circulatory microRNAs (miRNAs), which are small noncoding inhibitory RNAs, were shown to play a pivotal role in regulating pain processing within a wide range of experimental models and clinical pain disorders [31–34].

In this study, circulating miRNAs (miR-146a, miR-558, miR-155, and miR-124a) as biomarkers of back pain intensity were estimated using standard RT-PCR techniques. In subjects with moderate and severe LBP, we identified four circulating miRNAs, including decreased concentrations of miR-146a and miR-558 and increased concentrations of miR-155 and miR-124a, which were strongly associated with measures of BMI, WHtR, muscle function, pain intensity, malnutrition, PA score, and other LBP-related biomarkers such as vitamin D status, TNF-*α*, IL-6, and CRP levels, and markers of bone metabolism (s-Ca, s-BAP, s-OC, and s-NTX). The progression of pain intensity was shown to be linked with the aberrant expression of microRNAs [35–42]. Furthermore, significant changes in the expression of some miRNAs were reported in patients with peripheral inflammation, a nerve injury that drives severe pain [37–40]. These changes in miRNAs may induce alterations in the expression of some pain-associated genes, leading to an increase in neuronal excitability and behavioral pain hypersensitivity [38,40–42]. These studies indicate that miRNAs might be novel key players in the mechanisms underlying the development and maintenance of chronic pain.

Additionally, the stepwise regression analysis performed in this study revealed that miRNAs (miR-146a, miR-558, miR-155, and miR-124a), in association with vitamin D deficiency, bone markers, and other related LBP cofounders, were significantly associated with an incidence of LBP between 63.9% and 86.4% in older adults. Our data might support the possibility of applying microRNAs as therapeutics to alleviate established pain and as biomarkers in painful conditions (e.g., complex regional pain syndrome and fibromyalgia), as significantly reported in several clinical studies [43–45].

In summary, this study proposed that the regulation of miRNA expression may have potential prognostic value in subjects with LBP. Thus, the early detection of any changes in circulating miRNAs may play a promising role in identifying older subjects who may suffer from severe LBP.

## 5. Conclusions

In older adults with vitamin D deficiency, the severity of LBP was significantly associated with the expression of circulating miRNAs, adiposity, bone metabolism, inflammation, and muscle performance. In addition, this study revealed that miRNAs (miR-146a, miR-558, miR-155, and miR-124a), together with vitamin D deficiency, bone markers, and other related LBP cofounders, were significantly associated with an incidence of LBP between 63.9% and 86.4% in older adults. These results suggest the possibility of using microRNAs as therapeutics to alleviate established pain and as biomarkers in older adults with painful conditions.

## Figures and Tables

**Table 1:** Demographic, lifestyle, and other related biological parameters of the subjects according to LBP disability scores (OSW scores; *N* = 110).

	Normal (0%–20%) (*n* = 40; 36.4%)		Moderate-LBP (20%–40%) ^(b)^ (*n* = 35; 31.8%)		Severe-LBP (41%–60%) ^(b)^ (*n* = 35; 31.8%)	
	Male (*n* = 25, 62.5%)	Female (*n* = 15; 37.5%)	Male (*n* = 20, 57.14%)	Female (*n* = 15; 42.9%)	Male (*n* = 19, 54.3%)	Female (*n* = 16; 45.7%)
Age (year)	52.6 ± 3.6	52.3 ± 3.4	51.9 ± 4.1	51.6 ± 4.3	49.9 ± 3.7	49.4 ± 3.9

Anthropometry						
BMI	21.9 ± 3.6	22.8 ± 3.8	24.3 ± 4.6	26.9 ± 5.9^a^	26.1 ± 4.6^a^	28.1 ± 5.8^a^
WHtR	0.49 ± 0.11	0.51 ± 0.12	0.89 ± 0.15	0.96 ± 0.23^a^	1.2 ± 0.18^a^	1.31 ± 0.28^a^

Muscle mass						
MAC (in cm)	32.3 ± 1.5	33.6 ± 1.8	18.5 ± 2.9	16.3 ± 2.4^a^	13.8 ± 1.9	12.5 ± 1.4
TSF (in mm)	14.2 ± 3.2	15.3 ± 3.7	11.3 ± 4.6	10.4 ± 3.6^a^	9.6 ± 4.6	8.6 ± 5.1
MAMC (in mm)	21.9 ± 3.7	23.9 ± 4.1	15.9 ± 2.7	14.3 ± 1.9^a^	11.7 ± 2.1	9.8 ± 3.2

Diet measurements						
Diet score	18.3 ± 4.5	17.5 ± 4.7	11.5 ± 2.8	10.2 ± 2.6^a^	8.6 ± 5.1	6.8 ± 3.8^a^
Dietary vitamin D intake (IU/d)	318 ± 48	315 ± 46	211 ± 48	196 ± 42^a^	186 ± 61	136 ± 53^a^
Dietary Ca intake (mg/d)	1800 ± 67	1795 ± 62	854 ± 32	848 ± 38^a^	748 ± 49	711 ± 26^a^

SGA						
A (well nourished)	23 (92.0%)	13 (86.7%)	5 (25.0%)	2 (13.3%)^a^	2 (10.5%)	1 (6.25%)^a^
B (moderately malnourished)	2 (8.0%)	2 (13.3%)	12 (60.0%)	11 (73.3%)^a^	6 (31.6%)	6 (37.5%)^a^
C (severely malnourished)	0 (0%)	0 (0%)	3 (15%)	2 (13.3%)^a^	11 (57.9%)	8 (50.0%)^a^
Pain score	2.6 ± 1.3	2.9 ± 3.2	4.8 ± 3.7	6.9 ± 4.7^a^	8.9 ± 3.4	11.3 ± 2.8^a^
OSW score (0–100)	8.96 ± 3.6	11.3 ± 2.8	31.4 ± 9.1	38.2 ± 6.3^a^	54.9 ± 14.6	69.3 ± 18.5^a^

PA						
Total PA (counts/min)	3986 ± 250	3946 ± 210	2850 ± 125	2630 ± 112^a^	1800 ± 168	1685 ± 132^a^
MVPA (%)	96.5	94.3	68.9	58.3^a^	48.6	38.2^a^
Total energy (kcal/d)	6895 ± 520	6832 ± 415	2896 ± 185	2646 ± 175^a^	1578 ± 87	1356 ± 49^a^
Sun exposure (min/day)	25.4 ± 2.5	23.5 ± 1.6	18.8 ± 0.75	13.6 ± 0.64^a^	12.3 ± 0.65	9.5 ± 0.86^a^
TNF-*α* (pg/ml)	8.6 ± 1.4	8.9 ± 3.8	24.5 ± 7.5	29.7 ± 9.3^a^	37.9 ± 12.6	46.8 ± 13.6^a^
IL-6 (pg/ml)	3.8 ± 1.6	3.9 ± 2.8	8.5 ± 4.5	10.8 ± 3.7^a^	15.9 ± 6.8	19.9 ± 7.8^a^
CRP (mg/L)	5.1 ± 1.9	5.8 ± 2.8	12.4 ± 5.4	14.5 ± 6.7^a^	22.6 ± 8.1	25.8 ± 9.5^a^
Vitamin 25(OH)D (ng\mL)	48.7 ± 9.3	46.9 ± 8.6	24.9 ± 3.8	19.9 ± 5.8^a^	19.3 ± 4.6	12.8 ± 4.3^a^

Bone metabolism markers						
s-Ca (mmol/L)	2.8 ± 0.12	2.4 ± 0.18	2.6 ± 0.25	2.3 ± 0.18^a^	2.3 ± 0.62	1.9 ± 0.45^a^
s-BAP (U/L)	22.8 ± 5.7	21.9 ± 4.8	19.3 ± 4.3	14.8 ± 2.8^a^	15.6 ± 3.9	11.2 ± 2.5^a^
s-OC (ng/mL)	21.8 ± 4.3	20.6 ± 6.5	18.7 ± 5.7	12.9 ± 3.4^a^	16.6 ± 4.8	10.8 ± 3.5^a^
S-NTX (pg/mL)	480.6 ± 125.7	476.8 ± 115.4	365.8 ± 91.7	345.1 ± 65.2^a^	247.7 ± 42.7	198.3 ± 32.5^a^

All values were reported as mean ± SD or median (interquartile range) or percentage. Kruskal–Wallis one-way ANOVA and post hoc (Tukey's HSD) test were used to compare the mean values of the studied variables. Variables were considered significantly different at *p* < 0.05. ^a^*p* < 0.01 (males versus females in the same group), ^a^*p* < 0.001 (severe LBP or moderate LBP versus normal). Abbreviations: BMI: body mass index; WHtR: waist-to-height ratio; MVPA: moderate-to-vigorous physical activity; PA: physical activity; SGA: the Subjective Global Assessment; TNF-*α*: tumor necrosis factor-alpha; IL-6: interleukin-6; CRP: C-reactive protein; s-Ca: serum calcium; s-BAP: serum bone-specific alkaline phosphatase; s-OC: serum osteocalcin; S-NTX: serum C-terminal cross-linked telopeptide of type I collagen; MAC: mid-arm circumference in cm; TSF: triceps skinfold thickness in mm; MAMC: mid-arm muscle circumference in mm; LBP: low back pain; OSW: Oswestry Low Back Pain Disability Questionnaire.

**Table 2:** Correlation between circulating miRNAs concentrations and biological parameters in patients with low back pain (LBP) (*n* = 110).

Biological parameters	Whole cohort (qRT-PCR)^a^							
	Downregulated miRNAs				Upregulated miRNAs			
	miR-146a		miR-558		miR-155		miR-124a	
	R	P	R	P	R	P	R	P
Age	0.78	0.12	0.67	0.19	0.59	0.23	0.74	0.13
Gender	0.48	0.11	0.36	0.14	0.29	0.17	0.43	0.16
Adiposity (BMI/WHtR)	0.46	0.001	0.26	0.001	0.49	0.003	0.76	0.002
Muscle function	0.26	0.01	0.18	0.01	0.28	0.01	0.25	0.02
Malnutrition	0.18	0.01	0.21	0.01	0.31	0.01	0.29	0.01
PA score	0.31	0.01	0.45	0.01	0.39	0.01	0.67	0.01
Pain score	−0.48	0.001	−0.78	0.001	−0.58	0.001	−0.96	0.001
LBPscore (OSW score)	−0.86	0.001	−0.128	0.001	−0.38	0.001	−0.49	0.001
Inflammatory markers		0.001		0.001		0.001		0.001
TNF-*α* (pg/ml)	0.45		0.65		0.71		0.85	
IL-6 (pg/ml)	0.39		0.38		0.65		0.235	
CRP (mg/L)	0.75		0.86		0.48		0.345	
Bone metabolism		0.001		0.001		0.001		0.001
s-Ca (mmol/L)	−0.48		−0.86		−0.48		−0.87	
s-BAP (U/L)	−0.26		−0.56		−0.75		−0.59	
s-OC (ng/mL)	−0.36		−0.37		−0.18		−0.48	
S-NTX (pg/mL)	−0.58		−0.48		−0.56		−0.56	
Vitamin D status	0.65	0.001	0.38	0.001	0.89	0.002	0.78	0.001

^a^Data are R (spearman). Abbreviations: BMI: body mass index; WHtR: waist-to-height ratio; PA: physical activity; TNF-*α*: tumor necrosis factor-alpha; IL-6: interleukin-6; CRP: C-reactive protein; s-Ca: serum calcium; s-BAP: serum bone-specific alkaline phosphatase; s-OC: serum osteocalcin; S-NTX: serum C-terminal cross-linked telopeptide of type I collagen; LBP: low back pain; OSW: Oswestry Low Back Pain Disability Questionnaire, miR: microRNA.

**Table 3:** Stepwise multiple regression analysis for LBP predicted by adiposity, muscle function, PA score, malnutrition scores, markers of bone metabolism, inflammatory markers, vitamin D status, and miRNAs profile as the significant correlates in subjects with LBP (*n* = 110).

Variables	LBP disability scores (OSW scores; *N* = 110)					
	Normal (*n* = 40; 36.4%)		Moderate-LBP (*n* = 35; 31.8%)		Severe-LBP (*n* = 35; 31.8%)	
	R2 (*β*)^∗^	95% CI	R2 (*β*)^∗∗^	95% CI	R2 (*β*)^∗^	95% CI
Adiposity (BMI)	3.2 (0.51)	89 (75–96)	3.9 (0.49)	79 (69–95)	4.1 (0.47)	95 (89–100)
Vitamin D deficiency	6.5 (0.86)	95 (88–100)	7.1 (0.75)	96 (90–100)	7.6 (0.81)	97 (90–100)
Ca intake	3.1 (0.45)	90 (88–100)	2.8 (0.48)	96 (89–100)	1.6 (0.41)	91 (88–100)
Vitamin D intake	5.1 (0.36)	89 (86–100)	4.7 (0.75)	93 (89–100)	3.1 (0.67)	96 (89–100)

Muscle function						
MAC	2.3 (0.18)	88 (86–98)	1.8 (0.15)	78 (66–98)	1.4 (0.35)	87 (75–100)
MAMC	1.8 (0.14)	78 (69–100)	1.5 (0.18)	92 (69–100)	1.3 (0.32)	89 (85–100)
TSF	1.9 (0.19)	91 (89–100)	1.6 (0.25)	95 (89–100)	1.2 (0.36)	96 (89–100)
SGA	1.6 (0.21)	89 (87–100)	1.3 (0.29)	94 (87–100)	1.0 (0.31)	93 (87–100)
PA (min/wk)	4.2 (0.38)	88 (75–100)	3.5 (0.45)	89 (75–100)	2.9 (0.65)	91 (75–100)

miRNAs profile						
miR-146a	2.7 (0.85)	89 (86–100)	4.6 (0.75)	88 (86–100)	8.5 (0.65)	92 (86–100)
miR-558	3.3 (0.75)	87 (79–96)	3.9 (0.65)	89 (79–96)	5.2 (0.74)	95 (79–96)
miR-155	2.2 (0.59)	91 (88–100)	3.8 (0.49)	95 (88–100)	6.6 (0.56)	97 (88–100)
miR-124a	3.8 (0.46)	95 (90–100)	4.3 (0.42)	94 (90–100)	7.1 (0.48)	96 (90–100)

Inflammation profile						
TNF-*α* (pg/ml)	1.6 (0.26)	85 (79–96)	3.4 (0.28)	88 (79–96)	10.5 (0.36)	91 (79–96)
IL-6 (pg/ml)	2.8 (0.31)	90 (88–100)	3.9 (0.36)	95 (88–100)	7.3 (0.45)	96 (88–100)
CRP (mg/L)	1.8 (0.16)	88 (90–100)	3.2 (0.21)	91 (88–100)	6.9 (0.28)	94 (88–100)

Bone metabolism						
s-Ca (mmol/L)	3.6 (0.48)	95 (79–96)	2.9 (0.42)	89 (79–96)	2.5 (0.58)	95 (86–100)
s-BAP (U/L)	2.8 (0.35)	88 (79–96)	1.8 (0.32)	92 (79–96)	1.5 (0.38)	94 (79–100)
s-OC (ng/mL)	3.8 (0.24)	92 (88–100)	2.6 (0.29)	95 (88–100)	2.2 (0.32)	94 (88–100)
S-NTX (pg/mL)	5.8 (0.36)	86 (79–100)	4.3 (0.43)	89 (79–100)	3.9 (0.46)	91 (79–100)
ΣR2 (%)	63.9 (0.52)	98 (89–100)	66.9 (0.49)	96 (89–100)	86.4 (0.46)	95 (89–100)

*Note.* ^*∗*^ *p* < 0.01; ^*∗∗*^*p* < 0.001. Σ*R*2 = summation of cumulative values of *R* relating to studied variables. Abbreviations: CI, confidence interval; BMI, body mass index; MAC: mid-arm circumference in cm; TSF: triceps skinfold thickness in mm; MAMC: mid-arm muscle circumference in mm; SGA: the Subjective Global Assessment; PA: physical activity; miRNAs (small noncoding RNA molecules); OC: osteocalcin; s-BAP: serum bone alkaline phosphatase; s-Ca: serum calcium; S-NTX: serum C-terminal cross-linked telopeptide of type I collagen; TNF-*α*: tumor necrosis factor-alpha; IL-6: interleukin-6; CRP: C-reactive protein.

## Data Availability

All data generated or analyzed during this study are included within the manuscript. The data are also available from the corresponding author upon request.

## References

[1] Woo J., Leung J., Lau E. (2009). Prevalence and correlates of musculoskeletal pain in Chinese elderly and the impact on 4-year physical function and quality of life. *Public Health*.

[2] Edmond S. L., Felson D. T. (2000). Prevalence of back symptoms in elders. *The Journal of Rheumatology*.

[3] Jacobs J. M., Hammerman-Rozenberg R., Cohen A., Stessman J. (2006). Chronic back pain among the elderly: prevalence, associations, and predictors. *Spine*.

[4] March L. M., Brnabic A. J. M., Skinner J. C. (1998). Musculoskeletal disability among elderly people in the community. *Medical Journal of Australia*.

[5] Ulivieri F. M. (2007). Back pain treatment in post-menopausal osteoporosis with vertebral fractures. *Aging Clinical and Experimental Research*.

[6] Hübscher M., Vogt L., Schmidt K., Fink M., Banzer W. (2010). Perceived pain, fear of falling and physical function in women with osteoporosis. *Gait & Posture*.

[7] Liu-Ambrose T., Eng J. J., Khan K. M., Mallinson A., Carter N. D., McKay H. A. (2002). The influence of back pain on balance and functional mobility in 65- to 75-year-old women with osteoporosis. *Osteoporosis International*.

[8] Chou Y., Shih C., Lin J., Chen T., Liao C. (2013). Low back pain associated with sociodemographic factors, lifestyle and osteoporosis: a population-based study. *Journal of Rehabilitation Medicine*.

[9] Sinaki M., Pfeifer M., Preisinger E. (2010). The role of exercise in the treatment of osteoporosis. *Current Osteoporosis Reports*.

[10] Tarantino U., Baldi J., Celi M. (2013). Osteoporosis and sarcopenia: the connections. *Aging Clinical and Experimental Research*.

[11] Mayer T. G., Neblett R., Cohen H. (2012). The development and psychometric validation of the central sensitization inventory. *Pain Practice*.

[12] Solomonow M., Zhou B. H., Lu Y., King K. B. (2012). Acute repetitive lumbar syndrome: a multi-component insight into the disorder. *Journal of Bodywork and Movement Therapies*.

[13] Amirdelfan K., McRoberts P., Deer T. R. (2014). The differential diagnosis of low back pain: a primer on the evolving paradigm. *Neuromodulation: Technology at the Neural Interface*.

[14] Langevin H. M., Sherman K. J. (2007). Pathophysiological model for chronic low back pain integrating connective tissue and nervous system mechanisms. *Medical Hypotheses*.

[15] Ferreira G. D., Silva M. C., Rombaldi A. J., Wrege E. D., Siqueira F. V., Hallal P. C. (2011). Prevalência de dor nas costas e fatores associados em adultos do sul do Brasil: estudo de base populacional. *Brazilian Journal of Physical Therapy*.

[16] Chiuve S. E., Sampson L., Willett W. C. (2011). The association between a nutritional quality index and risk of chronic disease. *American Journal of Preventive Medicine*.

[17] Joshipura K. J., Hu F. B., Manson J. E. (2001). The effect of fruit and vegetable intake on risk for coronary heart disease. *Annals of Internal Medicine*.

[18] Riboli E., Norat T. (2003). Epidemiologic evidence of the protective effect of fruit and vegetables on cancer risk. *The American Journal of Clinical Nutrition*.

[19] Lotfi A., Abdel-Nasser A. M., Hamdy A., Omran A. A., El-Rehany M. A. (2007). Hypovitaminosis D in female patients with chronic low back pain. *Clinical Rheumatology*.

[20] Ruggiero C., Lattanzio F., Lauretani F., Gasperini B., Andres-Lacueva C., Cherubini A. (2009). Ω-3 polyunsaturated fatty acids and immune-mediated diseases: inflammatory bowel disease and rheumatoid arthritis. *Current Pharmaceutical Design*.

[21] Meleger A. L., Froude C. K., Walker J. (2014). Nutrition and eating behavior in patients with chronic pain receiving long-term opioid therapy. *PM&R*.

[22] Briggs M. S., Givens D. L., Schmitt L. C., Taylor C. A. (2013). Relations of C-reactive protein and obesity to the prevalence and the odds of reporting low back pain. *Archives of Physical Medicine and Rehabilitation*.

[23] Dowd J. B., Zajacova A. (2014). Long-term obesity and cardiovascular, inflammatory, and metabolic risk in U.S. adults. *American Journal of Preventive Medicine*.

[24] Galic S., Oakhill J. S., Steinberg G. R. (2010). Adipose tissue as an endocrine organ. *Molecular and Cellular Endocrinology*.

[25] Gebhardt K., Brenner H., Stürmer T. (2006). The course of high-sensitive C-reactive protein in correlation with pain and clinical function in patients with acute lumbosciatic pain and chronic low back pain-A 6 months prospective longitudinal study. *European Journal of Pain*.

[26] Huber F., Traber L., Roth H. J. (2003). Markers of bone resorption–measurement in serum, plasma or urine?. *Clinical Laboratory*.

[27] Calvo M. S., Eyre D. R., Gundberg C. M. (1996). Molecular basis and clinical application of biological markers of bone turnover. *Endocrine Reviews*.

[28] Crofton P. M., Kelnar C. J. (1998). Bone and collagen markers in paediatric practice. *International Journal of Clinical Practice*.

[29] de Ridder C. M., Delemarre-van de Waal H. A. (1998). Clinical utility of markers of bone turnover in children and adolescents. *Current Opinion in Pediatrics*.

[30] Szulc P., Seeman E., Delmas P. D. (2000). Biochemical measurements of bone turnover in children and adolescents. *Osteoporosis International*.

[31] Kynast K. L., Russe O. Q., Geisslinger G., Niederberger E. (2013). Novel findings in pain processing pathways: implications for miRNAs as future therapeutic targets. *Expert Review of Neurotherapeutics*.

[32] Kynast K. L., Russe O. Q., Möser C. V., Geisslinger G., Niederberger E. (2013). Modulation of central nervous system-specific microRNA-124a alters the inflammatory response in the formalin test in mice. *Pain*.

[33] Lutz B. M., Bekker A., Tao Y.-X. (2014). Noncoding RNAs. *Anesthesiology*.

[34] Niederberger E., Kynast K., Lötsch J., Geisslinger G. (2011). MicroRNAs as new players in the pain game. *Pain*.

[35] Fabbri M., Calin G. A. (2010). Epigenetics and miRNAs in human cancer. *Epigenetics and Cancer, Part A*.

[36] Farazi T. A., Spitzer J. I., Morozov P., Tuschl T. (2011). miRNAs in human cancer. *The Journal of Pathology*.

[37] Bai G., Ambalavanar R., Wei D., Dessem D. (2007). Downregulation of selective microRNAs in trigeminal ganglion neurons following inflammatory muscle pain. *Mol.Pain*.

[38] Sakai A., Saitow F., Miyake N., Miyake K., Shimada T., Suzuki H. (2013). miR-7a alleviates the maintenance of neuropathic pain through regulation of neuronal excitability. *Brain*.

[39] Sakai A., Suzuki H. (2013). Nerve injury-induced upregulation of miR-21 in the primary sensory neurons contributes to neuropathic pain in rats. *Biochemical and Biophysical Research Communications*.

[40] Zhao X., Tang Z., Zhang H. (2013). A long noncoding RNA contributes to neuropathic pain by silencing Kcna2 in primary afferent neurons. *Nature Neuroscience*.

[41] Shen W. S., Xu X. Q., Zhai N. N., Zhou Z. S., Shao J., Yu Y. H. (2017). Potential mechanisms of microRNA-141-3p to alleviate chronic nociceptive pain by downregulation of downstream target gene HMGB1.

[42] Sun E., Shi Y. (2015). MicroRNAs: small molecules with big roles in neurodevelopment and diseases. *Experimental Neurology*.

[43] Andersen H. H., Duroux M., Gazerani P. (2014 Nov). MicroRNAs as modulators and biomarkers of inflammatory and neuropathic pain conditions. *Neurobiology of Disease*.

[44] Park S. J., Cheon E. J., Kim H. A. (2013). MicroRNA-558 regulates the expression of cyclooxygenase-2 and IL-1*β*-induced catabolic effects in human articular chondrocytes. *Osteoarthritis and Cartilage*.

[45] Park C.-K., Xu Z.-Z., Berta T. (2014). Extracellular microRNAs activate nociceptor neurons to elicit pain via TLR7 and TRPA1. *Neuron*.

[46] Cole T. J., Bellizzi M. C., Flegal K. M., Dietz W. H. (2000). Establishing a standard definition for child overweight and obesity worldwide: international survey. *Br.Med.J.*.

[47] Ashwel M., Lejeune S., McPherson K. (1996). Ratio of waist circumference to height may be better indicator ofneed for weight management. *BMJ*.

[48] Lee R. D., Nieman D. C. (2003). Assessment of the hospitalized patient. *Nutritional Assessment*.

[49] Frisancho R. (1981). New norms of upper limb fat and muscle areas for assessment of nutritional status. *The American Journal of Clinical Nutrition*.

[50] Fritz J. M., Irrgang J. J. (2001). A comparison of a modified Oswestry low back pain disability questionnaire and the Quebec back pain disability scale. *Physical Therapy*.

[51] Fritz J. M., Clifford S. N. (2010). Low back pain in adolescents: a comparison of clinical outcomes in sports participants and nonparticipants. *Journal of Athletic Training*.

[52] Clifford S. N., Fritz J. M. (2003). Children and adolescents with low back pain: a descriptive study of physical examination and outcome measurement. *Journal of Orthopaedic & Sports Physical Therapy*.

[53] Detsky A., McLaughlin J. R., Baker J. (1987). What is subjective global assessment of nutritional status?. *Journal of Parenteral and Enteral Nutrition*.

[54] Detsky A. S., McLaughlin J. R., Baker J. P. (1987). What is subjective global assessment of nutritional status?. *JPEN*.

[55] Ciocîrlan M., Cazan A. R., Barbu M., Mănuc M., Diculescu M., Ciocîrlan M. (2017). Subjective global assessment and handgrip strength as predictive factors in patients with liver cirrhosis. *Gastroenterology Research and Practice*.

[56] Dixon W. G., Lunt M., Pye S. R. (2005 May). Low grip strength is associated with bone mineral density and vertebral fracture in women. *Rheumatology*.

[57] Otten J. J., Hellwig J. P., Meyers L. D. (2006). *Dietary Reference Intakes: The Essential Guide to Nutrient Requirements*.

[58] Harrell J. S., McMurray R. G., Baggett C. D., Pennell M. L., Pearce P. F., Bangdiwala S. I. (2005). Energy costs of physical activities in children and adolescents. *Medicine & Science in Sports & Exercise*.

[59] Trost S. G., Pate R. R., Sallis J. F. (2002). Age and gender differences in objectively measured physical activity in youth. *Medicine and Science in Sports and Exercise*.

[60] Alghadir A. H., Gabr S. A., Rizk A. A. (2018). Physical fitness, adiposity, and diets as surrogate measures of bone health in schoolchildren: a biochemical and cross-sectional survey analysis. *Journal of Clinical Densitometry*.

[61] Booth M. (2000). Assessment of physical activity: an international perspective. *Research Quarterly for Exercise and Sport*.

[62] Mäder U., Martin B. W., Schutz Y., Marti B. (2006). Validity of four short physical activity questionnaires in middle-aged persons. *Medicine & Science in Sports & Exercise*.

[63] Silva R. G., Fakhouri R., Nascimento T. V., Santos I. M., Barbosa L. M. (2008). Aspartate aminotransferase-to-platelet ration index for fibrosis and cirrhosis prediction in chronic hepatitis C patients. *The Brazilian Journal of Infectious Diseases*.

[64] Gabr S. A., Alghadir A. H. (2014). Prediction of fibrosis in hepatitis C patients: assessment using hydroxyproline and oxidative stress biomarkers. *Virusdisease*.

[65] Gabr S. A., Alghadir A. H. (2013). HCV genotypes and cellular immune response in correlation to liver fibrosis. *Journal of Pure and Applied Microbiology*.

[66] Fisher L., Fisher A. (2007). Vitamin D and parathyroid hormone in outpatients with noncholestatic chronic liver disease. *Clin Gastroenterol Hepatol*.

[67] Alghadir A., Gabr S., Al-Eisa E. (2017). Mechanical factors and vitamin D deficiency in schoolchildren with low back pain: biochemical and cross-sectional survey analysis. *Journal of Pain Research*.

[68] Alghadir A. H., Aly F. A., Gabr S. A. (2014). Effect of moderate aerobic training on bone metabolism indices among adult humans. *Pakistan Journal of Medical Sciences*.

[69] Hassannia T., GhaznaviRad E., Vakili R., Taheri S., Rezaee S. A. (2015). High prevalence of vitamin D deficiency and associated risk factors among employed women in a sunny industrial city. *International Journal for Vitamin and Nutrition Research*.

[70] Alghadir A. H., Gabr S. A., Al-Eisa E. (2015). Physical activity and lifestyle effects on bone mineral density among young adults: sociodemographic and biochemical analysis. *Journal of Physical Therapy Science*.

[71] Gorska-Ciebiada M., Saryusz-Wolska M., Borkowska A., Ciebiada M., Loba J. (2015). Serum levels of inflammatory markers in depressed elderly patients with diabetes and mild cognitive impairment. *PLoS One*.

[72] Al-Rawaf H. A. (2018). Circulating microRNAs and adipokines as markers of metabolic syndrome in adolescents with obesity. *Clinical Nutrition*.

[73] Erhardt J. G., Estes J. E., Pfeiffer C. M., Biesalski H. K., Craft N. E. (2004). Combined measurement of ferritin, soluble transferrin receptor, retinol binding protein, and c-reactive protein by an inexpensive, sensitive, and simple sandwich enzyme-linked immunosorbent assay technique. *The Journal of Nutrition*.

[74] Feng Y., Chen L., Luo Q., Wu M., Chen Y., Shi X. (2018). Involvement of microRNA-146a in diabetic peripheral neuropathy through the regulation of inflammation. *Drug Design, Development and Therapy*.

[75] Ortega F. J., Mercader J. M., Catalán V. (2013). Targeting the circulating microRNA signature of obesity. *Clinical Chemistry*.

[76] Kelly T. L., Berger N., Richardson T. L. (1998). DXA Body composition: theory and practice. *Applied Radiation and Isotopes*.

[77] Heneweer H., Staes F., Aufdemkampe G., van Rijn M., Vanhees L. (2011). Physical activity and low back pain: a systematic review of recent literature. *European Spine Journal*.

[78] Jacob T., Baras M., Zeev A., Epstein L. (2004). Physical activities and low back pain: a community-based study. *Medicine & Science in Sports & Exercise*.

[79] Wilmot E. G., Edwardson C. L., Achana F. A. (2012). Sedentary time in adults and the association with diabetes, cardiovascular disease and death: systematic review and meta-analysis. *Diabetologia*.

[80] Citko A., Górski S., Marcinowicz L., Górska A. (2018). Sedentary lifestyle and nonspecific low back pain in medical personnel in North-East Poland. *BioMed Research International*.

[81] Iwamoto J., Takeda T., Ichimura S. (2003). Urinary cross-linked N-telopeptides of type I collagen levels in patients with rheumatoid arthritis. *Calcified Tissue International*.

[82] Garnero P., Rousseau J.-C., Delmas P. D. (2000). Molecular basis and clinical use of biochemical markers of bone, cartilage, and synovium in joint diseases. *Arthritis & Rheumatism*.

[83] Takahashi M., Suzuki M., Naitou K. (1999). Comparison of free and peptide-bound pyridinoline cross-links excretion in rheumatoid arthritis and osteoarthritis. *Rheumatology*.

[84] Furumitsu Y., Inaba M., Yukioka K. (2000). Levels of serum and synovial fluid pyridinium crosslinks in patients with rheumatoid arthritis. *The Journal of Rheumatology*.

[85] van der Woude D., Young A., Jayakumar K. (2009). Prevalence of and predictive factors for sustained disease-modifying antirheumatic drug-free remission in rheumatoid arthritis: results from two large early arthritis cohorts. *Arthritis & Rheumatism*.

[86] Alghadir A. H., Gabr S. A., Al-Eisa E. S. (2016). Green tea and exercise interventions as nondrug remedies in geriatric patients with rheumatoid arthritis. *Journal of Physical Therapy Science*.

[87] Johansen J. V., Manniche C., Kjaer P. (2013). Vitamin D levels appear to be normal in Danish patients attending secondary care for low back pain and a weak positive correlation between serum level Vitamin D and Modic changes was demonstrated: a cross-sectional cohort study of consecutive patients with non-specific low back pain. *BMC Musculoskeletal Disorders*.

[88] Straube S., Moore A. R., Derry S., McQuay H. J. (2009). Vitamin D and chronic pain. *Pain*.

[89] Fernández-de-las-Peñas C., Hernández-Barrera V., Alonso-Blanco C. (2011). Prevalence of neck and low back pain in community-dwelling adults in Spain. *Spine*.

[90] Nagae M., Hiraga T., Wakabayashi H., Wang L., Iwata K., Yoneda T. (2006). Osteoclasts play a part in pain due to the inflammation adjacent to bone. *Bone*.

[91] Sinaki M. (1998). Musculoskeletal challenges of osteoporosis. *Aging Clinical and Experimental Research*.

[92] Sinaki M., Brey R. H., Hughes C. A., Larson D. R., Kaufman K. R. (2005). Balance disorder and increased risk of falls in osteoporosis and kyphosis: significance of kyphotic posture and muscle strength. *Osteoporosis International*.

[93] Zu B., Pan H., Zhang X.-J., Yin Z.-S. (2016). Serum levels of the inflammatory cytokines in patients with lumbar radicular pain due to disc herniation. *Asian Spine Journal*.

[94] Shamji M. F., Setton L. A, Jarvis W (2010). Proinflammatory cytokine expression profile in degenerated and herniated human intervertebral disc tissues. *Arthritis and Rheumatism*.

[95] de Queiroz B. Z., Pereira D. S., Lopes R. A. (2016). Association between the plasma levels of mediators of inflammation with pain and disability in the elderly with acute low back pain: data from the back complaints in the elders (BACE)-Brazil study. *Spine (Phila Pa 1976)*.

[96] Pedersen B. K., Steensberg A., Schjerling P. (2001). Muscle-derived interleukin-6: possible biological effects. *Journal of Physiology*.

[97] Shah J. P., Danoff J. V., Desai M. J. (2008). Biochemicals associated with pain and inflammation are elevated in sites near to and remote from active myofascial trigger points. *Archives of Physical Medicine and Rehabilitation*.

